# Differences in professional and personal lives between German female and male neurosurgeons — on behalf of the DGNC and EANS diversity committees

**DOI:** 10.1016/j.bas.2025.105882

**Published:** 2025-11-18

**Authors:** Tabea Miron, Dorothea Nistor-Gallo, Anna Lawson McLean, Ulrike Eisenberg, Jutta Krueger, Yu-Mi Ryang, Marion Hug, Marie-Therese Forster

**Affiliations:** a Department of Neurosurgery, Hospital Berlin-Buch, Schwanebecker Chaussee 50, 13125, Berlin, Germany; b Department of Neurosurgery, University Hospital Erlangen, Schwabachanlage 6, 91054, Erlangen, Germany; c Department of Neurosurgery, University Hospital Jena, Am Klinikum 1, 07747, Jena, Germany; d 12051, Berlin, Germany; e 22547, Hamburg, Germany; f Department of Neurology, Goethe University Hospital, Schleusenweg 2-16, 60528, Frankfurt am Main, Germany; g Department of Neurosurgery, Goethe University Hospital, Schleusenweg 2-16, 60528, Frankfurt am Main, Germany; h Department of Neurosurgery, Klinikum rechts der Isar, Technical University Munich, Ismaninger Str.,22, 81675, Munich, Germany

**Keywords:** Career, Diversity, Gender, Gender disparity, Gender equity, Neurosurgery

## Abstract

**Introduction:**

During the last two decades, the number of female neurosurgeons has increased, as has men's participation in childcare and household duties. Whether and to what extent professional and personal lives differ between male and female neurosurgeons in Germany remains unclear.

**Research question:**

This study investigates gender-specific differences in professional and personal lives among German neurosurgeons.

**Material and methods:**

An anonymous electronic 27-item questionnaire was distributed to all members of the German Neurosurgical Society (DGNC). Responses were stratified by gender and compared using Chi-square or Student's t-test.

**Results:**

Of 1558 neurosurgeons contacted, 290 (18.6 % response; 27.6 % female) participated (mean age 46.5 ± 11.4 years).

Female neurosurgeons were more likely to be single than their male colleagues (24.7 % vs. 6.5 %, p < 0.001), and more frequently had no children (54.4 % vs. 20.7 %, p < 0.001). Among respondents with children, 85.7 % of female neurosurgeons had stayed off work for childcare, while 92.6 % of male neurosurgeons indicated that their spouses had stayed at home (p < 0.001).

**Regarding career progression:**

women had significantly longer average training times compared with their male colleagues (7.6 ± 2.1 vs. 6.7 ± 1.2 years, p = 0.003). They encountered greater challenges experiencing inequity at work more frequently (60.8 % vs. 34.5 %, p < 0.001) and had more frequently the perception of obtaining a lower surgical case volume (39.7 % vs. 74.0 %, p < 0.001).

**Discussion and conclusion:**

Professional and personal lives differ significantly between male and female neurosurgeons in Germany. A paradigm shift in gender role attitudes and institutional measures is required to foster equity and improve career development opportunities in neurosurgery.

## Highlights

• Survey among German neurosurgeons shows gender-specific career disparities.
• Female neurosurgeons less often married and less frequently have children.
• Women more often report career breaks with negative impact on progression.
• Training duration significantly longer for female than for male neurosurgeons.
• Findings underline urgent need for strategies to foster gender equity.

## 1 Introduction

From the 1920s onwards, the development of neurosurgery in Germany was characterized by constant efforts to establish neurosurgery as an independent discipline. Alongside Walter Lehmann and Ludwig Guttmann, Alice Rosenstein (1898–1991) was not only a pioneer in the field, but also the first female neurosurgeon worldwide. Despite her groundbreaking work, being Jewish in Germany's 1930s lead her to emigrate to the United States, which did not stop her to rebuild her excellent academic reputation overseas. (Collmann et al.).

During the following decades, women only slowly began to establish themselves in neurosurgery and had to fight against various types of discrimination and exclusion. Although working conditions for women in neurosurgery have drastically improved since then, the continued underrepresentation of women among German neurosurgeons remains striking. In 2022, women made up 35 % of residents, 33.6 % of specialists and 23.1 % of senior physicians - and, most strikingly, only 9 % of neurosurgeons in leading positions (Forster et al., 2022).

The data collected in Germany are consistent with global trends. Neurosurgery, together with cardiothoracic surgery and orthopedics, remains one of the specialties with the lowest proportion of female faculty members (Newman et al., 2022) (Bennett et al., 2020). Moreover, female representation remains low on editorial boards of established neurosurgical journals, despite a significant increase in female authorship since 2009 (Bauman et al., 2022). Since then, the total number of both first and senior female authors has almost doubled. However, when analyzed by region, female authorship has only significantly increased in the US and Canada, but not in Europe (Pastor-et al., 2021). The trend of stagnation in Europe suggests that a glass ceiling has been reached, highlighting the need for further research to better understand this phenomenon, which may be linked to working and living conditions of neurosurgeons.

A survey of members of the European Association of Neurosurgical Societies showed that a significant proportion of neurosurgeons - particularly women - continue to experience gender inequality and discrimination in their workplace, which may lead to lower job satisfaction and, in consequence, lower productivity and quality of care (Zeitlberger et al., 2022). Furthermore, gender equity is not just an issue of justice and rights; it has also been shown to impact patient care (Tricco et al., 2021). Therefore, understanding gender-related factors affecting job satisfaction is essential to foster gender equity within the profession (Zeitlberger et al., 2022).

In this study we aimed to identify possible differences between female and male neurosurgeons in Germany concerning their professional and personal lives. Only by gaining this insight further steps will be successful towards a more equitable and meritocratic environment for neurosurgeons, regardless of gender.

## 2 Materials and methods

### 2.1 Data collection

An anonymous electronic survey consisting of 27 questions was sent to all members of the German Neurosurgical Society (DGNC) during the month of January 2021. The online survey was created using SurveyMonkey (SurveyMonkey Inc., San Mateo, CA, USA) and its design was guided by the principles outlined in the CHERRIES (Checklist for Reporting Results of Internet E-Surveys) guidelines (Eysenbach, 2004). Thus, 1558 neurosurgeons across all professional levels in hospitals of different ownership types, medical centers, and private practices throughout Germany were contacted. The survey link was distributed via the official DGNC mailing list, and one reminder email was sent two weeks after the initial invitation to maximize participation.

Responses received until the end of February 2021 were included in the final analysis. The survey contained key questions on demographics, marital status, childcare responsibilities, and surgical training, with a particular focus on identifying disparities across these areas. In this study, ‘staying home for childcare’ was defined as any period of work interruption for childcare purposes, including maternity leave, parental leave, or extended unpaid leave — the main options legally or commonly available in Germany. Equally, career interruptions, their reasons, and their consequences were inquired.

### 2.2 Statistical analysis

For data analysis, descriptive statistics were used. If applicable, survey responses were compared between groups using Chi-square and Fisher's exact test or the T-test, considering a p-value ≤0.05 statistically significant. Missing data per item were low (<4 %) and were excluded on a per-item basis when calculating percentages. All statistical analyses were performed using SPSS (version 26, IBM Corp.).

## 3 Results

A total of 290 neurosurgeons responded and completed the survey, resulting in a response rate of 18.6 %. In the following results, ∗ indicates comparisons between categorical variables using the Chi-square test, and+indicates comparisons of continuous variables using the independent-samples *t*-test.

### 3.1 Demographics

Basic demographics are listed in Table 1. Of all respondents 80 (27.6 %) self-identified as women and 210 (72.4 %) as men. Respondents worked in different professional settings, including 121 (41.7 %) and 118 (40.7 %) neurosurgeons working in university hospitals and non-university institutions, respectively, while 42 (14.5 %) neurosurgeons were established in private practice. For eight (2.8 %) respondents, no information on their professional setting was available. A huge number of respondents (n = 131; 45.1 %) held the status of a senior physician (also called senior consultant), and 27 (20.6 %) of these were female. Regarding all respondents' working positions and career levels, a significant difference in the distribution of female and male neurosurgeons was noted (p = 0.001∗). In contrast, academic ranks were similarly distributed between gender.

**Table 1:** Participant characteristics and gender differences.

	Total	Gender		p-value
		Female, n = 80	Male, n = 210	
Age, years (SD; range)	46.5 (11.4; 27–83)	42.6 (11.0; 27–80)	48.1 (11.2; 28–83)	0.000^b^
Working position and career levels, n (%)				0.000^a^
Private practice	42 (14.5)	8 (10.0)	34 (16.2)	
Hospital, senior physician	131 (45.1)	27 (33.8)	104 (49.5)	
Hospital, specialist	37 (12.8)	11 (13.8)	26 (12.4)	
Hospital, resident	44 (15.2)	27 (33.8)	17 (8.1)	
Other	36 (12.4)	7 (8.8)	29 (13.8)	

Academic rank, n (%)∗				
Professor	37 (12.7)	6 (7.5)	31 (14.8)	0.116^a^
Assistant professor	66 (22.8)	14 (17.5)	52 (24.8)	0.209^a^
Doctor	231 (79.7)	61 (76.3)	170 (81.0)	0.673^a^
None	59 (20.3)	19 (23.7)	40 (19.0)	0.673^a^

(The academic rank Professor or Assistant professor may be held together with a Doctorate; thus, multiple answers were possible.).

a Chi-square test was used for comparisons between categorical variables (e.g. gender, marital status, parenthood, working time).

b Independent-samples *t*-test was used for comparisons of continuous variables (e.g. duration of specialty training).

### 3.2 Marital status and parenthood

A significant difference was noted regarding respondents' marital status, as shown in Table 2. While 24.7 % of female neurosurgeons indicated to be single, only 6.5 % of their male counterparts did so (p < 0.001∗). Furthermore, 90.5 % of male, but only 72.7 % of female respondents stated to be married or in a committed partnership (p < 0.001∗). The parenthood gap between responding male and female neurosurgeon respondents was also significant. More specifically, 79 % of male, but only 46 % of female respondents affirmed to have children (p < 0.001∗). Accounting for the unbalanced distribution of male and female respondents among career levels, a similar level of parental roles between male and female respondents was only noted among residents (35.3 % of men vs. 30.8 % of women). Among specialists (who have completed their neurosurgical training but have still not obtained the title senior physician) and senior physicians, 73.1 % and 83.3 % of male neurosurgeons, but only respectively 54.5 % and 55.6 % of female neurosurgeons indicated to have children.

**Table 2:** Marital status, parenthood and working time.

	Total	Gender		p-value
		Female n (%)	Male n (%)	
Marital status				0.000^a^
Married/in relationship	238	56 (72.7)	182 (90.5)	
Divorced	8	2 (2.6)	6 (3.0)	
Single/living alone	32	19 (24.7)	13 (6.5)	

Parenthood (yes)	201	36 (45.6)	165 (79.3)	0.000^a^

Working time				0.001^a^
Full time	261	65 (82.3)	196 (95.1)	
Part-time	24	14 (17.7)	10 (4.9)	

If you have children - who stayed at home with them?				0.000^a^
My partner	142	5 (14.3)	137 (92.6)	
Me	41	30 (85.7)	11 (7.4)	

If you had children, would you stay at home with them? (yes)	147	51 (87.9)	96 (60.0)	0.000^a^

How long would you stay at home?				0.000^a^
1 month	9	0	9 (9.7)	
≤ 3 months	41	8 (15.7)	33 (35.5)	
≤ 6 months	27	9 (17.6)	28 (30.1)	
≤ 12 months	42	27 (52.9)	15 (16.1)	
≥ 12 months	15	7 (13.7)	8 (8.6)	

Percentages related to respective numbers of respondents.

^+^ Independent-samples *t*-test was used for comparisons of continuous variables (e.g. duration of specialty training).

a Chi-square test was used for comparisons between categorical variables (e.g. gender, marital status, parenthood, working time).

### 3.3 Working time and childcare

**Fig. 1:**
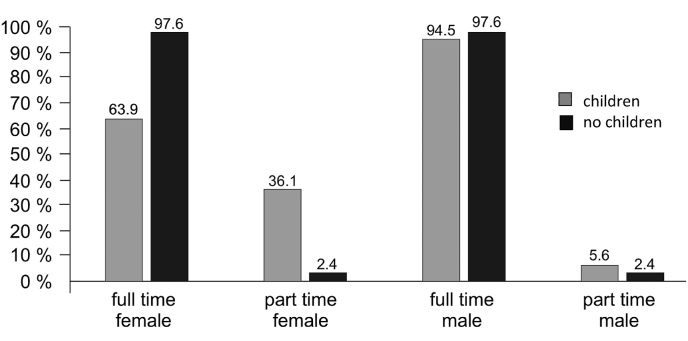
Employment status of German neurosurgeons by gender, with and without children.

Career flexibility and working times varied considerably between female and male respondents. Working part-time was indicated by 17.7 % of responding women, but only by 4.9 % of responding men. Correspondingly, among female neurosurgeons being mothers, 33 % worked part-time, while only 5 % of men having children did so, as depicted in Fig. 1.

**Fig. 2:**
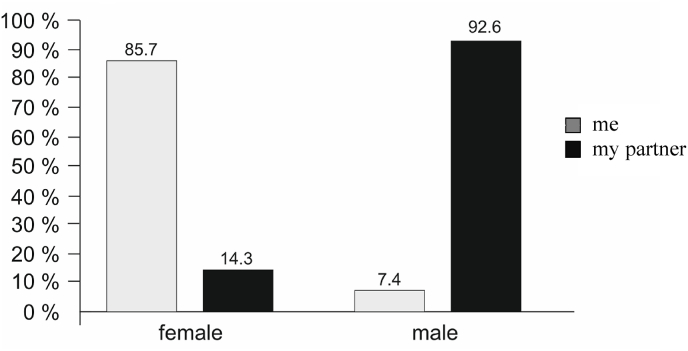
Childcare responsibilities. Bars indicating whether a female or male neurosurgeon or their partner have stayed off work for childcare. (female, *n* = 80; male, *n* = 210).

Among parental respondents, 85.7 % of female, but only 7.4 % of male neurosurgical counterparts had stayed home for childcare, as shown in Fig. 2.

When asked whether they would hypothetically choose to stay home for childcare, 87.9 % of women expressed willingness to do so, compared to just 60 % of men (p < 0.001∗). Similarly, the hypothetical duration of those inclined to take child-care related leaves differed significantly: 52.9 % of women were willing to stay at home for up to 12 months, compared to only 16.1 % of men (p < 0.001∗). Most men (33.5 %) would stay at home no longer than 3 or 6 months (35.5 % and 30.1 % of male respondents, respectively).

### 3.4 Specialty training, career breaks and effects of career interruptions

Responding neurosurgical specialists reported an average specialty training time of 6.9 ± 1.5 years. The duration of specialty training differed significantly between female and male neurosurgeons, coming up to 7.6 ± 2.1 and 6.7 ± 1.2 years, respectively (p = 0.003^+^). This difference remained unchanged even after adjusting for parenthood, with average training times of 7.6 ± 1.8 years for mothers and 7.6 ± 2.5 years for childless female neurosurgeons, respectively. Among all respondents, 42.9 % had changed hospitals during their residency. Training interruptions were reported by 40 (50.6 %) female and 50 (24.8 %) male respondents (p < 0.001∗). Although childcare was the most common reason for career interruption reported by both, women and men (64.7 % and 47.8 % of those having interrupted their career), men cited more often 'other' reasons for the interruption than their female counterparts (39.1 % vs. 20.6 %; not statistically significant). Male neurosurgeons were significantly more likely to view the effects of an interruption positively compared to their female colleagues (p = 0.001∗). While 60.5 % of men stated that the break had an overall positive effect on their career overall, only 22.9 % of women shared that view. Conversely, 77.1 % of female respondents felt that the break negatively impacted their careers. Moreover, after an interruption of training, significantly fewer women (52.5 %) than men (96 %) returned to work full-time (p < 0.001∗).

### 3.5 Feeling of discrimination

Perceived injustice during training was reported by 60.8 % of female respondents and 34.5 % of male respondents (p < 0.001∗). Among women who felt unfairly treated 77.1 % attributed the perceived disadvantage to their gender, compared to just 5.9 % of men. By contrast, 11.8 % of these men rather felt discriminated against due to their language, while only 4.2 % of women who felt unfairly treated reported the same (p < 0.001∗). Similar patterns were observed when respondents were asked for the surgical case volume during training, as presented in Table 3.

**Table 3:** Specialty training, career breaks and their effects and the feeling of discrimination.

	Gender		p-value
	Female, n (%)	Male, n (%)	
Have you ever taken a career break? (yes)	40 (50.6)	51 (24.8)	0.001^a^
Yes …			0.268^a^
… for maternal/paternal leave	22 (64.7)	22 (47.8)	
… for caring of relatives	1 (2.9)	1 (2.2)	
… caused by illness	2 (5.9)	1 (2.2)	
… for further education or science	2 (5.9)	4 (8.7)	
… due to other reasons	7 (20.6)	18 (39.1)	

Have you returned in full-time after the career break? (yes)	20 (52.6)	48 (96)	0.000^a^

Had this break a positive influence on your career? (yes)	8 (22.9)	26 (60.5)	0.001^a^

Have you ever experienced inequity at work? (yes)	48 (60.8)	70 (34.5)	0.000^a^
Yes …			0.000^a^
… due to gender	37 (77.1)	4 (5.9)	
… due to language	2 (4.2)	8 (11.8)	
… due to the color of my skin	0	1 (1.5)	
… due to other reason	9 (18.8)	55 (80.9)	

Is/was your surgical case volume the same as of colleagues of the same career level? (yes)	31 (39.7)	151 (74.0)	0.000^a^
No …			0.000^a^
… due to gender	30 (68.2)	4 (7.7)	
… due to language	1 (2.3)	6 (11.5)	
… due to the color of my skin	0	1 (1.9)	
… due to other reason	13 (29.5)	41 (78.8)	

Time of training for board-certification, years (SD; range)	7.6 (2.1; 5–16)	6.7 (1.2; 5–13)	0.003^b^

Percentages related to respective numbers of respondents.

a Chi-square test was used for comparisons between categorical variables (e.g. gender, marital status, parenthood, working time).

b Independent-samples *t*-test was used for comparisons of continuous variables (e.g. duration of specialty training).

Of note, male respondents most frequently cited “other reasons” – rather than gender or language – for perceived unequal treatment (n = 55, 80.9 % of males responding “yes”) or for an imbalanced surgical case volume (n = 41, 78.8 % of males responding “yes”). Thematic analysis of free-text responses revealed several perceived causes, including preferential treatment or behavior by supervisors (e.g., favoritism or lack of support, 30.9 %, n = 17), structural or institutional factors such as rigid hierarchies or poor team dynamics (16.4 %, n = 9), origin, dialect or language issues (12.7 %, n = 7) or personality traits or interpersonal mismatch (10.9 %, n = 6). A detailed breakdown is provided in Supplementary Table 1.

## 4 Discussion

Despite the rising number of women in surgical specialties and pleas for gender equality in medicine during recent years, gender-related differences still exist according to the pertaining literature as well as the present study. This applies not only to working conditions, but also regarding work-life balance.

Several studies have documented that female surgeons are not only less likely to be married but are also less likely to have children (Bucknor et al., 2018; Steklacova et al., 2017; Dyrbye et al., 2012; Baptiste et al., 2017; Lambrianou et al., 2022; Rangel et al., 2021; Okoshi et al., 2016; Gadjradj et al., 2020; Granek et al., 2024; Li et al., 2024)*.* By postponing pregnancy because of surgical training, female surgeons more frequently use assisted reproductive technology, they have higher rates of obstetric complications and more often must face infertility compared to partners of their male colleagues (Li et al., 2024). Indeed, pregnancy loss may be found in up to 42 % of female surgeons, a rate that is twice as high as the rate of the general population (Rangel et al., 2021). In a recent study involving 5692 US surgical residents, 22.3 % of men but only 10.2 % of women reported on at least one pregnancy during their training (Li et al., 2024).

Focusing on the field of neurosurgery, in which women account for the minority of the medical workforce (Lulla et al., 2021), parenthood seems even less balanced among genders. In two surveys among European neurosurgeons more than half of female neurosurgeons were childless compared to only one third of their male colleagues (Steklacova et al., 2017; Lambrianou et al., 2022). The present study confirms these data, with 54.4 % of women and 20.7 % of men indicating being childless. Interestingly, within the subgroup of German neurosurgical residents a smaller gender gap of parenthood was noted (35.3 % of men vs. 30.8 % of women). This contrasts not only to previous data but also data from the US, where 31.1 % of male but only 6.1 % of female neurosurgeons aged 30 years or less indicated giving birth to a child (Gadjradj et al., 2020). One reason for these diverging numbers around parenthood might be found in the variation of maternity leave and parental leave policies across countries. Thus, Debbie et al. reported on only 45 of 835 US surgical residents who had experienced pregnancy during training and who had taken parental leave for longer than 7 weeks (Li et al., 2024). In Germany, female workers are not allowed to work within the first 8 weeks after delivery while receiving full salary, and they may benefit from combined paid parental leave (with the payment based on the monthly net disposable income) and protection against unfair dismissal up to 14 months after their child's birth. Consequently, more than 90 % of mothers take advantage of parental leave during their child's first year of life (Germany Mikrozensus, 2019). German fathers, however, only rarely make use of parental leave, with only a quota coming up to 14.2 % seizing that opportunity.

Within the German neurosurgical workforce, the relation of genders taking parental leave does not differ from the general population. While 85.7 % of female neurosurgeons indicate having taken time off to stay with their children, 92.6 % of male neurosurgeons reported that it had been their partners who took leaves of absence to take care of their children. However, if they became parents today, 60 % of responding male neurosurgeons claim that they would stay at home with their child at present, with most of them intending to take 3 or 6 months off from work. Although this is shorter than the duration that most female neurosurgeons would choose themselves, male neurosurgeons seem increasingly willing to support the trend towards egalitarian domestic responsibilities.

Nevertheless, up to now, German male neurosurgeons who have taken a career break did so for child-rearing purposes only in half of the cases. In many instances, they interrupted their careers for research projects, a fellowship or other reasons. This also explains why 60.5 % of them considered their career breaks to have a positive impact on their professional life, while 77.1 % of female neurosurgeons who took a temporary leave from work viewed its effect as negative for their career. This impression of female neurosurgeons is commonly referred to as “motherhood penalty”, subsuming females' delay or renunciation of pregnancy, their lower wages, their fewer promotion opportunities and persistent and widespread maternity discrimination (Di et al., 2024; Correll et al., 2007; Yu and Hara, 2021). A recent study on female cardiologists revealed that women not only tend to work in subspecialties compatible with family life, but also frequently must face prejudice and harassment based on their status as mothers (Ruzycki et al., 2022). Moreover, once mothers, women are often perceived as less productive due to the neccessity to balance family and work responsibilities, while fatherhood is typically associated with greater responsibility and a stronger motivation to work and be the sole provider (Yu and Hara, 2021). Although latest evidence shows that the productivity of female surgeons does not significantly decrease after maternity, prejudice surrounding motherhood appear to persist, driven by biased assumptions (Chen et al., 2022).

In neurosurgery, a plethora of international studies published during the last years have demonstrated, that women are still not only significantly underrepresented, but also confronted with discrimination and prejudice regardless of their parental status (Zeitlberger et al., 2022). Unsurprisingly, also within the present study 60.8 % of responding female neurosurgeons report on their experience of discrimination in contrast to 34.5 % of their male colleagues. While these women attributed their feelings of unequal treatment primarily to their gender (77.1 %), it is noteworthy that men who felt discriminated against rarely attributed discrimination to their gender (5.9 %) but primarily cited other reasons for their discrimination, such as language (11.8 %), or structures of preferential treatment or behavior by superiors and their origin, cultural and social background.

This observation underlines the importance of examining how gender inequalities intersect with other social categories such as race and class. The concept of intersecting identities - where multiple identity axes and power dynamics intersect - is increasingly recognized in interdisciplinary research and provides a useful framework for understanding the complexity of perceived discrimination at work (Bennamate, 2024). Current research is examining the extent to which intersectional discrimination operates within medicine, particularly in highly specialized surgical disciplines such as neurosurgery.

Finally, it is worth noting that female neurosurgeons report specialty training durations that are, on average, 11 months longer than those of their male counterparts, regardless of parenthood, highlighting a significant disparity in training opportunities for women in German neurosurgery. These differences are not due to part-time work or career interruptions, but likely reflect structural inequalities: women often spend more time on ward duties and patient consultations and receive less operative exposure and mentorship. As progression in German neurosurgery largely depends on individual supervisors and operative opportunities, these disparities can delay the acquisition of required surgical competencies, highlighting the need for structured and equitable training programs for all trainees.

A significant proportion of respondents reported interruptions in their specialist training, primarily due to childcare, but also for research projects or health reasons (50.6 % of women vs. 24.8 % of men). The numbers show that women interrupt more often, as they are still more responsible for care work overall, and that the system generally allows interruptions, which creates a certain flexibility for German neurosurgeons.

The lack of equal training opportunities, role models, promotions and sponsorship, as well as difficulties in balancing family and work, have been cited as key contributing factors to attrition rates among female surgeons, which, in neurosurgery, have been shown to be more than three times higher than for male colleagues (Richter et al., 2020; Lynch et al., 2014; Stienen et al., 2016; Gelman et al., 2024; Chen et al., 2023). Considering recent evidence that patients treated by female surgeons spend significantly more days alive and at home after surgery and exhibit lower complication and mortality rates at 90 days and 1 year compared to those operated on by male surgeons, the retention, encouragement and promotion of women in the field of surgery is essential (Wallis et al., 2022; Heybati et al., 2024; Saka et al., 2024). The creation and maintenance of a diverse and representative surgical workforce may not only improve patient outcome, thereby lowering healthcare costs, but also foster creativity and innovation and enhance patient satisfaction and adherence (Gomez and Bernet, 2019; Rosenkranz et al., 2021).

However, as demonstrated in the present study, in historically male dominated medical specialties such as neurosurgery a transformation towards gender equity remains slow and is hindered by deep-rooted cultural norms and systemic barriers, limiting progress. How may these challenges be overcome?

Acknowledging gender-related disparities and understanding one's own implicit bias is the first important step towards change. This is true for everyone in the team, with departmental leadership being crucial to develop a culture that accepts and welcomes diversity not only in gender, but also regarding culture, age, abilities and expertise. Equally, mentorship not only of those resembling oneself is needed. On an institutional level, the creation of an environment encouraging pregnancy, supporting surgeons of both genders in balancing work and private life, allowing flexible working conditions and job-sharing paradigms will contribute to a modern healthcare system.

### 4.1 Study limitations

This study has the following limitations. First, the response rate of 18.6 % of German neurosurgeons might not exactly represent the complete neurosurgical workforce. As with all studies relying on a survey and self-reported data, it is possible that only neurosurgeons with a particular interest responded, potentially introducing response bias. To address this, respondent demographics were compared to overall DGNC membership data (gender, career level, sector), showing a similar distribution. However, the number of 290 respondents including 80 female neurosurgeons may also be seen as a strength considering the relatively small neurosurgical workforce. Moreover, the distribution of respondents closely mirrors the German neurosurgical workforce (Forster et al., 2022).

Second although the data were collected during the COVID-19 pandemic, we do not expect this to have influenced the responses, since participants reflected on their overall career experiences and neurosurgeons remained clinically active throughout the pandemic. Furthermore, given that the structural and professional framework of neurosurgical training and working conditions in Germany has remained largely unchanged since then, we believe that the data have not lost their relevance or validity. A follow-up survey is planned to assess potential longitudinal changes in the coming years.

Third, to allow statistical computations and comparability, subjective opinions were only asked additionally for some topics and could not all be taken into consideration for this article. Finally, the study design was cross-sectional, collecting data at a single time point and not allowing conclusions about causality. Thus, further evaluations over time are needed to support our findings. Finally, although we attempted to adjust for potential confounders, small subgroup sizes and interdependencies between variables precluded a fully adjusted analysis, which may have led to underestimation of potential interactions and residual confounding.

## 5 Conclusion

The present study highlights that gender equity in German neurosurgery remains far from being achieved. Women more often forgo having children, interrupt their careers, or work part-time for childcare, illustrating the difficult balance between neurosurgery and family life. The time-intensive nature of the job leaves little opportunity for care work, so men rarely participate to the same extent, leaving women to shoulder the main burden due to traditional gender roles. All neurosurgeons, regardless of gender, face challenges in balancing professional and personal responsibilities. Women tend to experience greater career setbacks when raising children, whereas men often advance further in their careers but have less time for family.

The underlying causes are multifaceted and complex, ranging from systemic barriers, cultural norms and values to personal biases and gender disparity in overall society. However, research has consistently shown the benefits of a diverse workforce. In healthcare, gender equity and diversity are associated with improved decision-making, higher quality of patient care, reduced healthcare costs and improved patient satisfaction (Wallis et al., 2022; Heybati et al., 2024; Saka et al., 2024; Gomez and Bernet, 2019; Simms, 2013). As such, surgical communities, including the German Neurosurgical Society, should prioritize the development of a diverse surgical workforce – a goal that will require concerted effort. While first steps have been taken, much more remains to be done in order to change mentalities and structural barriers. The time for action is now.

## Compliance with ethical standards

Ethics statement: The manuscript does not contain clinical studies or patient data.

## Funding

No funding was received for this work.

## Conflict of interest

The authors have no personal, financial, or institutional interests in any of the material presented in this article.
