# Supplementary material for: Differences in professional and personal lives between German female and male neurosurgeons — on behalf of the DGNC and EANS diversity committees
Source: Brain Spine. 2025 Nov 18;5:105882. doi: 10.1016/j.bas.2025.105882 (PMC12731929; doi:10.1016/j.bas.2025.105882)
Supplement: Multimedia component 1 [file mmc1.pdf]

Fragen zur Person

Geschlecht: ○ männlich ○ weiblich ○ anderes

Alter: ☐ Jahre

Bundesland: ○ BB ○ BE ○ BW ○ BY ○ HB ○ HE ○ HH ○ MV
○ NI ○ NW ○ RP ○ SH ○ SL ○ SN ○ ST ○ TH

Tätigkeitsfeld: ○ Uni-Klinik ○ nicht-universitäres Krankenhaus ○ Praxis ○ anderes

Position: ○ Oberarzt/ Oberärztin ○ Facharzt/ Fachärztin ○ Assistenzarzt/ Assistenzärztin
○ selbständig ○ anderes

Beschäftigungs-ausmaß: ○ Vollzeit ○ Teilzeit → ☐ Stunden pro Woche

Familienstand: ○ ledig/ allein lebend ○ verheiratet/ in Beziehung ○ geschieden

Haben Sie Kinder? ○ Nein ○ Ja → Ist Ihr (jüngstes) Kind < 6 Jahre?
○ Nein ○ Ja

Kinderbetreuung: Wer blieb für die Kinderbetreuung wie lange zu Hause?

☐ Ich ☐ Monate

☐ Partner/ Partnerin ☐ Monate

Wenn Sie Kinder hätten, würden Sie für die Kinderbetreuung zu Hause bleiben? Wenn ja, wie lange?

○ Nein ○ Ja → ○ 1 Monat ○ < 3 Monate
○ ≤ 6 Monate ○ ≤ 12 Monate

Fragen zur beruflichen Laufbahn

In welchem Jahr haben Sie Ihre Facharzt-Ausbildung begonnen? ☐

In welchem Jahr haben Sie Ihre Facharzt-Ausbildung abgeschlossen? ☐

Haben Sie während Ihrer Ausbildung die Klinik gewechselt? ○ Nein ○ Ja

Haben Sie Ihre berufliche Tätigkeit unterbrochen? ○ Nein ○ Ja, für ☐ Monate

Grund der Unterbrechung: ☐ Krankheit ☐ Kinderbetreuung ☐ Pflege von Angehörigen ☐ Aus-/ Fortbildung ☐ anderes

In welchem Umfang kehrten Sie initial in das Berufleben zurücK?

○ Vollzeit ○ Teilzeit

Hatte die Tätigkeitsunterbrechung positive oder negative Auswirkungen auf Ihre Karriere?

○ positiv ○ negativ

Sind Sie promoviert? ○ Nein ○ Ja → ☐ Jahr der Promotion

Streben Sie eine Promotion an? ○ Nein ○ Ja

Sind Sie habilitiert? ○ Nein ○ Ja → ☐ Jahr der Habilitation

Streben Sie eine Habilitation an? ○ Nein ○ Ja

Führen Sie den Titel Professor? ○ Nein ○ Ja → ☐ Jahr der Professur

Haben/ hatten Sie den Eindruck, während Ihrer Ausbildung ungerecht behandelt zu werden?

○ Nein ○ Ja

Falls Sie den Eindruck haben/ hatten, während Ihrer Ausbildung ungerecht behandelt zu werden, was war Ihrer Meinung nach der Grund?

☐ Sprache ☐ Hautfarbe ☐ Geschlecht

☐ Anderes: ______________________________
