# Supplementary material for: Differences in professional and personal lives between German female and male neurosurgeons — on behalf of the DGNC and EANS diversity committees
Source: Brain Spine. 2025 Nov 18;5:105882. doi: 10.1016/j.bas.2025.105882 (PMC12731929; doi:10.1016/j.bas.2025.105882)
Supplement: Multimedia component 2 [file mmc2.docx]

| Thematic Category | Examples | n (%) |
| --- | --- | --- |
| Preferential treatment or behavior of supervisors | “Chief physician favored certain individuals”, “lack of support”, “sympathy/antipathy”, “leadership style” | 17 (30.9%) |
| Structural or institutional barriers | “Hospital hierarchy”, “internal politics”, “poor rotation system”, “lack of training opportunities” | 9 (16.4%) |
| Origin, language, background | “Foreign background”, “accent/dialect”, “race and gender” | 7 (12.7%) |
| Personality or team fit | “Character traits”, “lack of opportunism”, “disciplinary conflicts” | 6 (10.9%) |
| Workload and operative access | “Too much OR time, no time for research”, “few surgical cases”, “inequitable distribution” | 9 (16.4%) |
| Networking or influence (“Vitamin B”) | “No connections”, “colleagues with prominent relatives” | 2 (3.6%) |
| Family-related constraints | “Parental leave”, “childcare responsibilities” | 2 (3.6%) |
| Unclear or diffuse explanations | “Not specified”, “unclear”, “personal tensions” | 3 (5.5%) |

Supplemental Table 1. Thematic categorization of free-text responses by male neurosurgeons citing “other reasons” for perceived discrimination or unequal surgical case volume, n=55
